# Biogenic copper oxide nanoparticles from *Bacillus coagulans* induced reactive oxygen species generation and apoptotic and anti-metastatic activities in breast cancer cells

**DOI:** 10.1038/s41598-023-30436-y

**Published:** 2023-02-24

**Authors:** Masoumeh Dolati, Farzaneh Tafvizi, Masoud Salehipour, Tahereh Komeili Movahed, Parvaneh Jafari

**Affiliations:** 1 Department of Biology, Parand Branch, Islamic Azad University, Parand, Iran; 2 Cellular and Molecular Research Center, Qom University of Medical Sciences, Qom, Iran; 3 Microbiology Department, Faculty of Science, Arak Branch, Islamic Azad University, Arak, Iran

**Keywords:** Cell biology, Nanoscience and technology

## Abstract

The present study examined the anticancer capabilities of *Bacillus coagulans* supernatant-produced copper oxide nanoparticles (BC-CuONPs) on MCF-7 and SKBR3 cancer cells. The X-ray diffraction, ultraviolet–visible spectroscopy, Fourier-transform infrared spectroscopy, X-ray photoelectron spectroscopy, transmission electron microscopy, field-emission scanning electron microscopy, energy-dispersive X-ray, dynamic light scattering, and zeta potential techniques were used to characterize BC-CuONPs. This study also investigated the cellular and molecular processes of NPs’ anti-proliferative and apoptotic properties on human breast cancer cells and compared them to the commercial pharmaceutical tamoxifen. The size of the spherical NP was from 5 to 47 nm with negative zeta potential. The MTT results showed the great cytotoxic effect of BC-CuONPs against breast cancer cells. The BC-CuONPs inhibited the growth of breast cancer cells in a time- and dose-dependent manner. The up-regulation of BCL2-associated X (*BAX*)*,* cyclin dependent kinase inhibitor 1A (*P21*), Caspase 3 (*CASP3*), and Caspase 9 (*CASP9*), the down-regulation of BCL2 apoptosis regulator (*BCL2*), Annexin V-FITC/propidium iodide, and reactive oxygen species (ROS) generation results suggested that BC-CuONPs had a significant apoptotic impact when compared to the control. Scratch tests and vascular endothelial growth factor receptor gene (*VEGF*) down-regulation demonstrated that BC-CuONPs had anti-metastatic activity. The cell cycle analysis and down-regulation of Cyclin D1 (*CCND1*) and cyclin dependent kinase 4 (*CDK4*) revealed that cancer cells were arrested in the sub-G1 phase. Finally, the results showed that the secondary metabolites in the supernatant of *Bacillus coagulans* could form CuONPs, and biogenic BC-CuONPs showed anti-metastasis and anticancer properties on breast cancer cells while having less adverse effects on normal cells. Therefore, the synthesized CuONPs using *B. coagulans* supernatant can be shown as a potential candidate for a new therapeutic strategy in cancer management.

## Introduction

Breast cancer is the most frequent malignancy in women, responsible for further than 1 out of every 10 new cancer cases annually^1^. It is also the world’s second leading trigger of mortality from cancer among women. Although the disorder occurs globally, its incidence, mortality, and overall survival differ significantly worldwide, which might be attributable to a variety of variables, such as the organization of genetic variations in a population, lifestyle, and ecology^2^. Women can be classified depending on their risk factors for breast cancer, which can help improve risk-free procedures and build tailored breast cancer diagnostic strategies^3^. Conventional breast cancer therapies rely on neoplasm features, including clinical stage, histopathologic characteristics, and biomarker analysis at first^4^. Surgery, chemotherapy, radiation therapy, endocrine therapy, and targeted therapy are the most common treatments for breast cancer.

Breast-conserving surgery is a popular therapeutic option for site-specific breast cancer^5^. Furthermore, conventional therapies might have a negative impact on the experience of the lives of breast cancer individuals. As a result, patients with breast cancer must be treated with a less harmful and destructive strategy.

MCF-7 and SKBR3 are breast cancer cell lines. MCF-7 cells that express estrogen, progesterone, and androgen receptors have the morphology of breast epithelium. SKBR3 cells were isolated from a patient with adenocarcinoma, and in terms of cell surface receptors, they express human epidermal growth factor receptor 2 (HER2) and are estrogen and progesterone negative. HER2 is a growth factor receptor and plays a role in physiological cell growth and differentiation. This protein increases the proliferation and invasion of healthy tissues by signaling pathways related to cancer^6,7^.

In the past several years, one of the promising therapeutic approaches has been nanotechnology. Nanotechnology provides the targeted and precise delivery of chemotherapeutics to malignant cells and neoplasms, the direction of surgical removal of malignancies, and the enhancement of the therapeutic potential of radiation-based and other existing therapeutic methods^8^. An important property of nanoparticles (NPs) is attributed to the small size of 1–100 nm, distinct shape, high surface area to volume ratio, and solubility^9^. Metal NPs have been found to serve a positive and significant function in cancer treatment by improving selectivity, gene silencing, and pharmaceutical delivery^10^.

Previous investigations have shown that copper oxide nanoparticles (CuONPs) are extremely harmful to a variety of cancer cells, including hepatocarcinoma, breast cancer, cervical carcinoma, lung carcinoma, and pancreatic cancer^11–16^. The anticancer effect of CuONPs is achieved by an elevation in reactive oxygen species (ROS) generation and oxidative stress, which induces deoxyribonucleic acid (DNA) breakage, enhanced death receptor overexpression, and, finally, apoptosis-driven cell death^17^. As a result, it can be considered a medical representative in cancer therapy^18^. Apart from the pro-angiogenic properties of CuONPs, several experiments have also shown that these NPs have anti-angiogenic action and can prevent the migration of vascular endothelium, thereby inhibiting angiogenesis^19,20^. Furthermore, CuONPs have antibacterial and antifungal action and might be used as a contrast agent in multimodal imaging^21,22^.

Biological approaches that are harmless, economical, and ecologically friendly have gained popularity in the last several years, as contrasted with physicochemical NP production procedures. The synthesis of metallic NPs can be accomplished using fully “green” approaches by employing an ecologically compatible solvent solution with eco-friendly reducing and stabilizing agents. Several routes have been identified for the biogenic or biological fabrication of NPs from metal ion salts^23–25^. The NPs are synthesized using microorganisms, fungi, herbal extracts, and marine algae^6,20,26–29^. The reduction of metal ions from diverse biomolecules, such as enzymes/proteins, polysaccharides, amino acids, and vitamins, is the underlying mechanism in the biogenesis of NPs^30^.

Bacteria have well-documented strategies for surviving in unpleasant environments, such as excessive levels of poisonous metal, by converting harmful metal ions into non-toxic forms (e.g., metal sulfide/oxides). It should be noted that all these pathways play an important part in the biogenesis of NPs. Furthermore, NPs biosynthesis by bacteria has various advantages over other species, such as easy culture, extracellular NPs creation in mild experimental conditions (e.g., temperature and pH), and being inexpensive and time-saving. *Bacillus* species have been demonstrated to be a promising option for the biosynthesis of different metallic NPs, particularly CuONPs^31^.

Elbeshehy et al.^32^ synthesized and characterized silver nanoparticles (AgNPs) using *Bacillus pumilus*, *Bacillus persicus*, and *Bacillus licheniformis*. In another study, *Bacillus subtilis* was applied for the biogenic synthesis of AgNPs^33^. In a survey, the *Bacillus coagulans* bacteria was also used for the synthesis of Fe_3_O_4_ NPs as stabilizing and bio-reducing agents^34^. However, there is no study on the biosynthesis of CuONPs using *Bacillus coagulans*.

Several microorganisms, including *Bacillus* spp., are known for their ability to synthesize NPs. Nevertheless, using *Bacillus* spp., able to carry out a reliable biosynthesis of NPs with certain properties, such as having a particular composition and size, is at the forefront of nanotechnological research. For the first time, the present study describes *Bacillus coagulans* as a great source for the reduction of CuONPs, with important anticancer and apoptotic activities. The synthesized NPs were broadly characterized using physical and chemical methods, including spectrophotometry, electron microscopy, energy-dispersive X-ray spectroscopy (EDX), and Fourier-transform infrared spectroscopy (FTIR). The anticancer activity of the NPs was assessed against breast cancer cell lines, and the expression of pro-apoptotic and anti-apoptotic genes was evaluated using quantitative real-time reverse transcription polymerase chain reaction (PCR); however, their apoptotic and cell cycle arrest potential were in vitro evaluated regarding both MCF-7 and SKBR-3 cell lines.

## Materials and methods

### Materials

*Bacillus coagulans* strain (GBI-30, 6068) was purchased from Ganeden, Inc. (the USA). SKBR3 and MCF-7 breast cancer cell lines and the human foreskin fibroblasts (HFF) normal cell line were obtained from the National Center for Genetic and Biological Resources of Iran. CuSO_4.5_H_2_O, tamoxifen, and MTT (3-[4,5-dimethylthiazol-2-yl]-2,5-diphenyltetrazolium bromide) were purchased from Sigma-Aldrich, Germany. Trypan blue and sodium bicarbonate (NaHCO_3_) were purchased from Merck, Germany. Biological reagents used for cell culture experiments, such as Dulbecco’s Modified Eagle Medium (DMEM; with 4.0 mM l-glutamine and sodium pyruvate), RPMI 1640, 0.25% Trypsin-EDTA (1X), and phosphate-buffered saline (PBS) pH 7.4, were purchased from Gibco, the UK. Moreover, fetal bovine serum (FBS) was purchased from Gibco, the USA. Penicillin/streptomycin (10,000 U/mL) was purchased from Sigma-Aldrich, the USA. RNX-Plus kit for ribonucleic acid (RNA) extraction and complementary DNA (cDNA) synthesis kit (RevertAid TM Kit) were obtained from CinnaGen, Iran, and Thermo Scientific Fermentas, the USA, respectively. The Annexin V-FITC/propidium iodide (PI) apoptosis kit was purchased from MabTag GmbH, Germany.

### Bacterial culture and storage

The culture of *Bacillus coagulans* and preparation of cell-free supernatant were carried out according to a previous study^35^*.* Briefly, *B. coagulans* (GBI-30, 6068) were bought as lyophilized from Ganeden, Inc. (the USA). The lyophilized bacterial powder was incubated in Tryptic Soy Broth (TSB) for 10 min at 80 °C until the spores’ germination. The TSB medium was then cultured for 24 h in a shaker incubator at 30 °C and 150 rpm. To keep the bacteria, it was cultured for 18 h in de Man, Rogosa, and Sharpe (MRS) broth medium before being incubated at 30 °C. Afterward, 25% glycerol was administered and aliquoted in 1.5 mL Eppendorf tubes. The Eppendorf tubes were chilled and kept at − 70 °C (Thermo Fisher Scientific, USA).

To prepare the cell-free supernatant, 50 mL of MRS with the bacterial stock was administered. Subsequently, the mixture was incubated for 18 h in the same manner. To finish the synthesis of metabolites, 10% of the overnight culture was inoculated into an MRS broth medium and cultured for 72 h. Centrifugation was carried out at 6000 rpm (Hettich, Germany), at room temperature, for 20 min to obtain the bacterial culture supernatant. Then, 100 mL of the culture medium was thickened with a freeze dryer to about 20 mL and stored in a refrigerator^35^.

### BC-CuONPs biosynthesis

In this study, 10 mL of thickened bacterial supernatant was added to 100 mL of 5 mM copper sulfate solution (CuSO_4.5_H_2_O, 99.9% purity, Merck, Germany) with a temperature of 80 °C, and the pH was regulated to 7 with NaOH and incubated for 24 h on a shaker heater in the dark. As a negative control, MRS broth culture medium without supernatant with copper sulfate and copper sulfate solution without supernatant were used. In both controls, no color change was observed. The first step in confirming the formation of CuONPs is the change in the color of the solution. Slowly, the accumulation of brownish-black particles appeared at the bottom of the Erlenmeyer flask by changing the color from light blue to dark green. After 24 h, the solution containing NPs was poured into 50 mL falcons and centrifuged at 14,000 rpm for 30 min to separate the NPs. The NP pellet was redissolved in 1 mL of sterile water and centrifuged at 14,000 rpm for 15 min (three times for washing). Then, the NP was dried in the incubator and weighed; subsequently, the dilution was prepared^27,36^.

### Characterization of BC-CuONPs

The crystallographic structure of BC-CuONPs was determined by an X-ray diffractometer (XRD) (Bruker AXS model D8 Advance using Cu Ka radiation within the range of 2θ = 10°–90°)^37,38^. The chemical features of BC-CuONPs were recorded by FTIR (Bruker Tensor 27, Biotage, Germany; scanning range of 4000–400 cm^−1^; resolution of 4 cm^−1^ in the transmittance mode). The dried powder of biosynthesized CuONPs powder was mixed with potassium bromide and pressed into the disc for FTIR analysis^39,40^. The chemical composition of oxide CuONPs was analyzed by a X-ray photoelectron spectroscopy (XPS) analyzer (BESTEC, Germany)^41,42^. The spectroscopic analysis of BC-CuONPs was performed within the range of 200–800 nm using an ultraviolet–visible (UV–Vis) spectrophotometer (UV 1601, Shimadzu Corp, Japan)^43,44^. One drop of the BC-CuONPs solution was placed on the copper-coated carbon grades after the ultrasonic treatment of BC-CuONPs for transmission electron microscopy (TEM) analysis to determine the size and morphology of BC-CuONPs; nevertheless, the EDX spectrum revealed the elemental constituent of the prepared sample (Leo 906, Zeiss100 KV model, Germany)^39,45^. For field-emission scanning electron microscopy (FESEM) preparation, the samples were spread on a silica sheet and dried at room temperature. Then, they were coated with gold in a sputter coating unit to analyze their size and morphology (ZEISS, Sigma VP model, Germany)^46^. The particle size (hydrodynamic size distribution), polydispersity index (PDI), and zeta potential of CuONPs were determined by Malvern Instruments Nano Zetasizer (Worcestershire, the UK)^37^.

### Cell culture and cytotoxicity assay

The cell lines were cultivated in RPMI 1640 and DMEM with 10% FBS, 100 μg/mL of streptomycin, and 100 units/mL of penicillin in a 5% carbon dioxide (CO_2_) cell incubator at 37 °C. The cytotoxicity of BC-CuONPs and tamoxifen against MCF-7 and SKBR3 breast cancer cells and HFF normal cells was evaluated via MTT assay. For this method, the cells were quantified before being placed onto 96-well plates at a frequency of 5000 cells/well and incubated for 24 h in a CO_2_ incubator at 37 °C. In 96-well plates, both cancerous and normal cells were treated within a range of 3.125–100 μg/mL of BC-CuONPs and tamoxifen. The MTT stock solution (5 mg/mL in PBS) was established, and 20 μL of MTT solution was administered to each treatment well, proceeded by a 4-h incubation at 37 °C and 5% CO_2_. The resultant violet formazan crystals were then dissolved in dimethyl sulfoxide, and the optical density of each well was determined at 570 nm using a microplate reader (Synergy/HTX, Bio Tek, Germany)^47,48^. The influence of biosynthesized NPs and drugs on cells was quantified as a percentage of cell viability relative to the control, which was calculated using the following formula:

$$\% {\text{Cell}}\;{\text{viability}} = \left( {{\text{mean}}\;{\text{absorbance}}\;{\text{of}}\;{\text{treated}}\;{\text{cells}}/{\text{mean}}\;{\text{absorbance}}\;{\text{of}}\;{\text{control}}\;{\text{cells}}} \right) \times {1}00$$

### In vitro apoptosis/necrosis assay

Flow cytometry was used to investigate apoptosis/necrosis cells labeled with the fluorescein-conjugated-Annexin V/PI test. In each 6-well plate, 1 × 10^5^ MCF-7, SKBR3, and HFF cells were seeded independently. The cells were then treated individually with the half maximal inhibitory concentration (IC_50_) concentrations of BC-CuONPs and tamoxifen in a 37 °C incubator for 48 h before being examined using the Annexin V/PI assay (Apoptosis detection kit, MabTag GmbH, Germany), according to the kit protocols. The cells that were not treated served as a control. Subsequently, flow cytometry was used to assess the amounts of necrotic/apoptotic cells^49^ (Attune NxT Flow Cytometer, Thermo Fisher Scientific, the USA). Data analysis was carried out using FlowJo software (Version 7.6.1).

### Cell cycle assay

The PI staining was used to evaluate cell growth. The cell cycle stage was determined by examining the DNA content, where the interaction of PI with DNA is proportionate to the DNA concentration. The cells were seeded in 6-well plates with complete media at a density of 1 × 10^5^ cells/well and incubated overnight at 37 °C. The cells were washed and plated three times in PBS. The cells were then treated for 48 h in full media with BC-CuONPs and tamoxifen. Following incubation, the cells were detached and fixed with 70% cold ethanol overnight at 4 °C before being stained with 500 µL of PI solution (50 µg/ml) at room temperature in the dark condition for 10 min. Finally, flow cytometry was used to examine them (Attune NxT Flow Cytometer, Thermo Fisher Scientific, the USA)^50,51^.

### Gene expression analysis

BCL2 associated X (*BAX*), BCL2 apoptosis regulator (*BCL2*)*,* caspase 3 (*CASP3*), caspase 9 (*CASP9*)*,* cyclin-dependent kinase inhibitor 1A (*P21* or *CDKN1*)*,* cyclin-dependent kinase 4 (*CDK4*)*,* cyclin D1 (*CCND1*)*,* cyclin B1 (*CCNB1*)*,* cyclin E1 (*CCNE1*)*,* vascular endothelial growth factor (*VEGF*), vascular endothelial growth factor receptor (*VEGFR*), and beta-actin (*ACTB*) messenger RNAs (mRNAs) expression levels were quantified using SYBR Green real-time quantitative PCR in MCF-7 and SKBR3 cells. The studied cell lines were seeded into 6-well plates (1 × 10^5^ cells/well) and incubated for 48 h, following treatment with the IC_50_ concentration of BC-CuONPs and tamoxifen for another 48 h. Total cellular RNA was isolated from cells using the RNA-isolation kit, as directed by the manufacturer. High-quality RNA was extracted for cDNA synthesis utilizing the RevertAid TM Kit in accordance with the manufacturer’s instructions. The primers utilized for real-time PCR are shown in Table 1. The target gene expression was investigated via a real-time PCR technique (StepOnePlus, Applied Biosystems, USA). Each PCR amplification reaction was carried out in a 25 μL reaction mixture comprising 12.5 μL Master Mix (2 ×) (Amplicon, Denmark), 1 μL of each primer (0.4 μM), 1 μL cDNA (100 ng), and 9.5 μL diethyl pyrocarbonate water. After denaturation at 95 °C for 10 min, 40 cycles were followed by 95 °C, 55–62.5 °C, and 72 °C for 20, 40, and 40 s in PCR cycling conditions, respectively. All reactions for the examined genes were repeated three times, and the melting curve was obtained at 60 and 95 °C for 1 min and 15 s, respectively. The comparative threshold cycle (Ct) was used to evaluate gene expression levels. The average Ct value of *ACTB* as a reference gene was then deducted from the average Ct value of the targeted genes to get the ΔCt values for each sample. Finally, the target/reference control gene expression ratio (ratio: 2^−ΔΔCt^) was computed^52^.

**Table 1:** The utilized primers in this research.

Gene	Reverse
β-actin *(ACTB)*	Forward: 5′-AGGTCTTTGCGGATGTCCACGT-3′ Reverse: 5′-CACCATTGGCAATGAGCGGTTC-3′
BCL2-associated X *(BAX)*	Forward: 5′-AGCTTCTTGGTGGACGCATC-3′ Reverse: 5′-TTGCTTCAGGGTTTCATCCAG-3′
BCL2 apoptosis regulator *(BCL2)*	Forward: 5′-CAGCCAGGAGAAATCAAACAGAG-3′ Reverse: 5′-TGTGGATGACTGAGTACCTGAACC-3′
Caspase 9 *(CASP9)*	Forward: 5′-TTAGTTCGCAGAAACGAAGC-3′ Reverse: 5′-CATATGATCGAGGACATCCAG-3
Caspase 3 *(CASP3)*	Forward: 5′-ACAGTCCAGTTCTGTACCACG-3′ Reverse: 5′-AGAGGGGATCGTTGTAGAAGTC-3′
Cyclin B1 *(CCNB1)*	Forward: 5′-AATAAGGCGAAGATCAACATGGC-3′ Reverse: 5′-TTTGTTACCAATGTCCCCAAGAG-3′
Cyclin D1 *(CCND1)*	Forward: 5′-AAGTTGTTGGGGCTCCTCAG-3′ Reverse: 5′-CAGATCATCCGCAAACACGC-3′
Cyclin E1 *(CCNE1)*	Forward: 5′-AGTTTGGGTAAACCCGGTCAT-3′ Reverse: 5′-GCCAGCCTTGGGACAATAATG-3′
Vascular endothelial growth factor *(VEGF)*	Forward: 5′-GCTTGTCACATCTGCAAGTACG-3′ Reverse: 5′-TGTCTAATGCCCTGGAGCCT-3′
Vascular endothelial growth factor receptor *(VEGFR)*	Forward: 5′-AAA CCC ATT TGG CAC ATC TGT-3′ Reverse: 5′-ATC ATT CCG AAG CAA GGT GTG-3′
Cyclin dependent kinase inhibitor 1A *(P21)*	Forward: 5′-CCCTTCAAAGTGCCATCTGT-3′ Reverse: 5′-GCTTCATGCCAGCTACTTCC-3′
Cyclin dependent kinase 4 *(CDK4)*	Forward: 5′-CCA ACA CTC CAC ATG TCC AC-3′ Reverse: 5’′-CAT CGT TCA CCG AGA TCT GA-3′

### Reactive oxygen species detection assay

TPR-ROS Test Kit (TEB PAZHOUHAN RAZI, Iran) is a fluorometric assay that employs the ROS-sensitive probe DCFH-DA. Once DCFH-DA has spread into cells, the ester hydrolysis enzyme hydrolyzes it into a non-fluorescent molecule (DCFH) and quickly oxidizes it to bright green fluorescence DCF (dichlorofluorescein). The magnitude of the generated fluorescence is related to the quantity of ROS in cellular systems and can be measured by enzyme-linked immunosorbent assay (Bio Tek, the USA) (ex 485/em 535). Briefly, the most susceptible MCF-7 and SKBR3 cell lines were treated with the IC_50_ concentrations of BC-CuONPs and tamoxifen for 48 h. The cells were washed in 100 µL buffer before 60 min of incubation at 37 °C with 100 µL DCF. Then, 100 μL of R3 stimulator was added to the designated positive control wells (H_2_O_2_) and incubated for 20 more minutes at 37 °C in the dark. The DCF staining buffer was carefully removed, and 100 μL of the prepared test buffer was added. A microplate reader was used to quantify the fluorescence intensity at an excitation wavelength within the range of 480–500 nm and an emission wavelength within the range of 510–550 nm.

### Scratch assay

In order to analyze cell migration, MCF-7 cancer cells were planted at 1 × 10^5^ cells/well in 6-well plates and incubated until they achieved 70% confluence. The samples were scratched with a 200 μL pipette tip to generate a lesion, and the cells were then washed repeatedly with serum-free culture to exclude floating cells. The medium was changed with new serum-free media. For 36 h, the cells were cultured in the media containing the IC_50_ concentrations of BC-CuONPs and tamoxifen. The cells were further washed with PBS, fixed, and observed under a microscope^53^.

### Statistical analysis

GraphPad Prism software (version 8.0.2) was used for statistical analysis and curve fitting. All the findings from three different experiments were expressed as mean ± standard deviation. A t-test and two-way analysis of variance were performed to determine the significance level of variations between groups (*P < 0.05, **P < 0.01, ***P < 0.001, ****P < 0.0001).

### Informed consent

All authors consent to the publication of this study.

## Results and discussion

### Nanoparticles characterization

**Figure 1:**
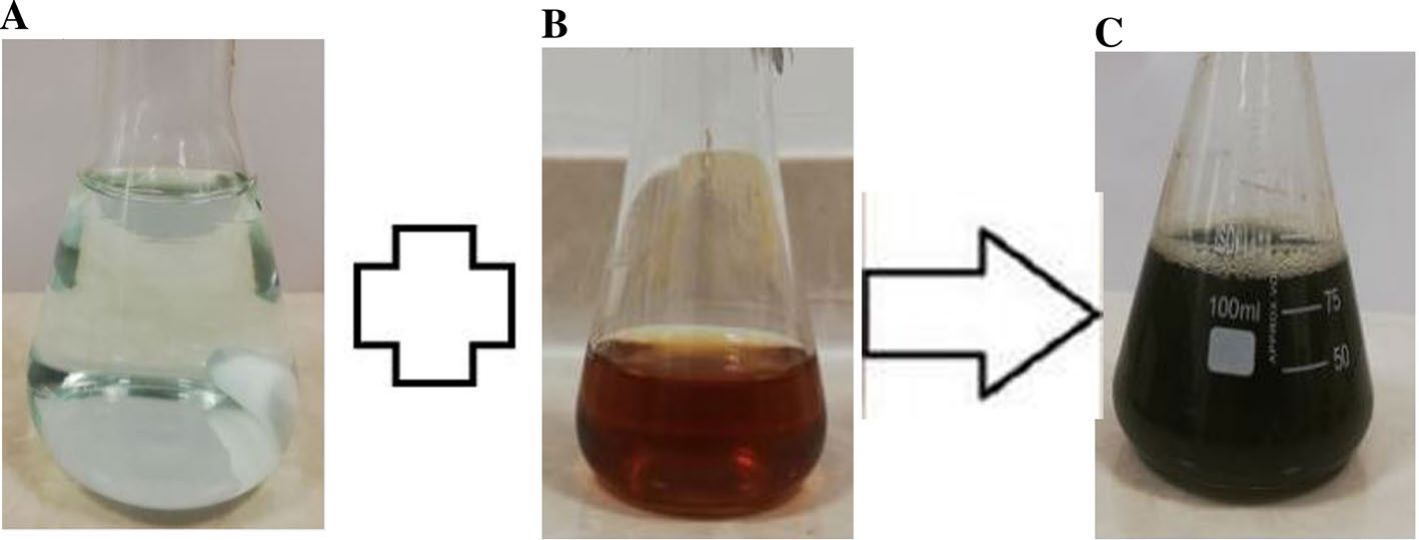
Color change assay for validation of CuONPs synthesis. (**A**) CuSo4 solution, (**B**) Supernatant of *Bacillus coagulans*, (**C**) biosynthesized CuONPs using *Bacillus coagulans* (BC-CuONPs).

**Figure 2:**
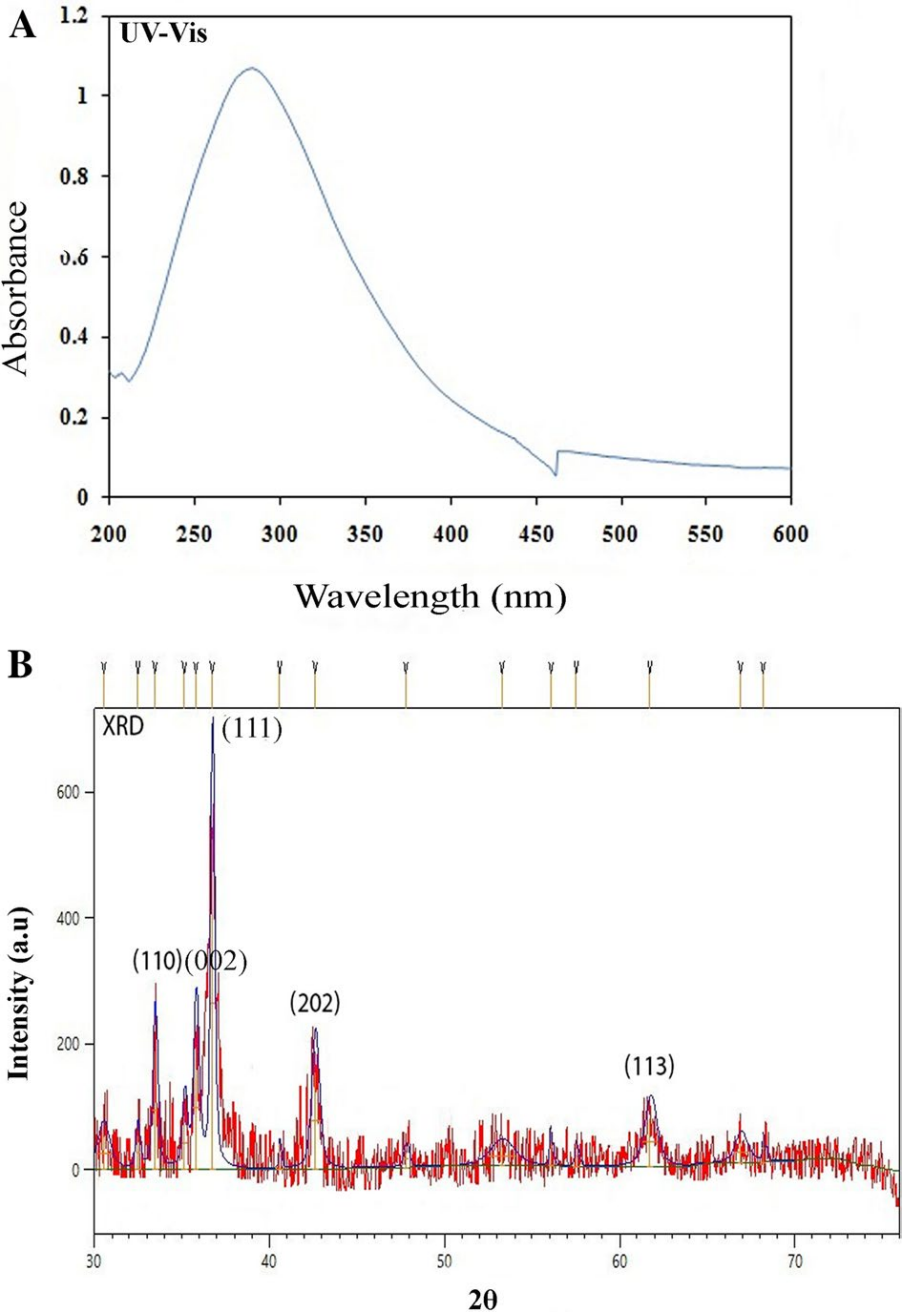
(**A**) UV–visible spectrum, (**B**) XRD graph of BC-CuONPs.

In the current study, CuONPs were strongly synthesized using *B. coagulans* supernatant, and color change was observed from light blue (copper (II) sulfate) to dark green, indicating the reduction of Cu^2+^ ions and synthesis of CuONPs (Fig. 1). The UV–Vis spectrum of BC-CuONPs was performed within the range of 200–600 nm (Fig. 2A). The maximum absorption in the synthesized sample was observed at 280 nm. A previous study also showed the excitonic absorption peak in the UV–Vis spectra at 280 nm^54^.

This study used the XRD method to analyze the crystalline structure of BC-CuONPs (Fig. 2B). The five main characteristic XRD peaks were observed in 2θ values of 33.45, 35.76, 36.73, 42.57, and 61.64 degrees, which correspond to (110), (002), (111), (202), and (113) planes of face‐centered cubic (FCC) copper oxide crystalline structure, respectively. This result is in line with the standard information published for the XRD pattern of CuONPs. As stated by similar studies, the XRD method revealed that biogenic CuONPs are crystalline with FCC structure^55,56^.

**Figure 3:**
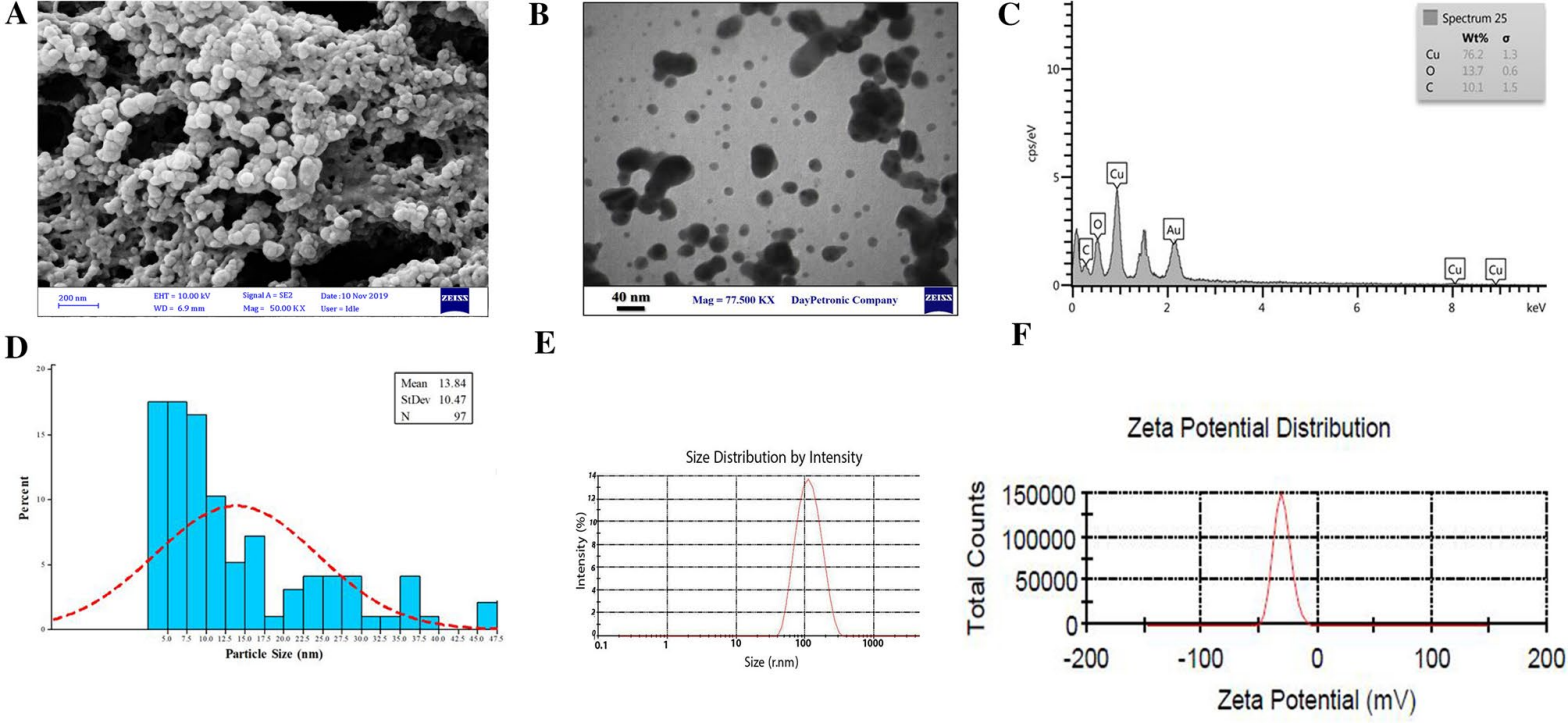
(**A**) FE-SEM, (**B**) TEM, (**C**) EDS, (**D**) Particle size, (**E**) DLS and (**F**) Zeta-potential of BC-CuONPs, Zeta potential and DLS values were obtained after 5 running cycles.

The size of the BC-CuONPs was estimated in the range from 5 to 47 nm with mean size 13.84 nm with a spherical shape, according to the TEM and FESEM images (Fig. 3A,B,D). In the EDX analysis for the elemental composition of the BC-CuONPs, the weight percentage of copper and oxygen was reported as 76% and 13.7%, respectively (Fig. 3C). Dynamic light scattering (DLS) analysis also indicated that the average colony size of CuONPs synthesized using *B. coagulans* supernatant was estimated at 102 nm (Fig. 3E). The PDI was reported to be 0.15, indicating that the synthesized NPs were uniform. The zeta potential was used to calculate the effective electric charge on the surface of BC-CuONPs. The zeta potential value of − 30 mV represented that the BC-CuONPs had perfect stability with a negative charge^57^ (Fig. 3F).

The recent investigations indicated that the particle size of CuONPs using *Pseudomonas fluorescens* and *Azadirachta indica* was within the range of 20–80 nm, which showed good agreement with the current study’s results^58,59^. The measured size in the DLS technique was much larger than the obtained size from TEM. The reason is that DLS only measures the hydrodynamic diameter of NPs, and they are suspended in contact with the other available atoms^60^.

**Figure 4:**
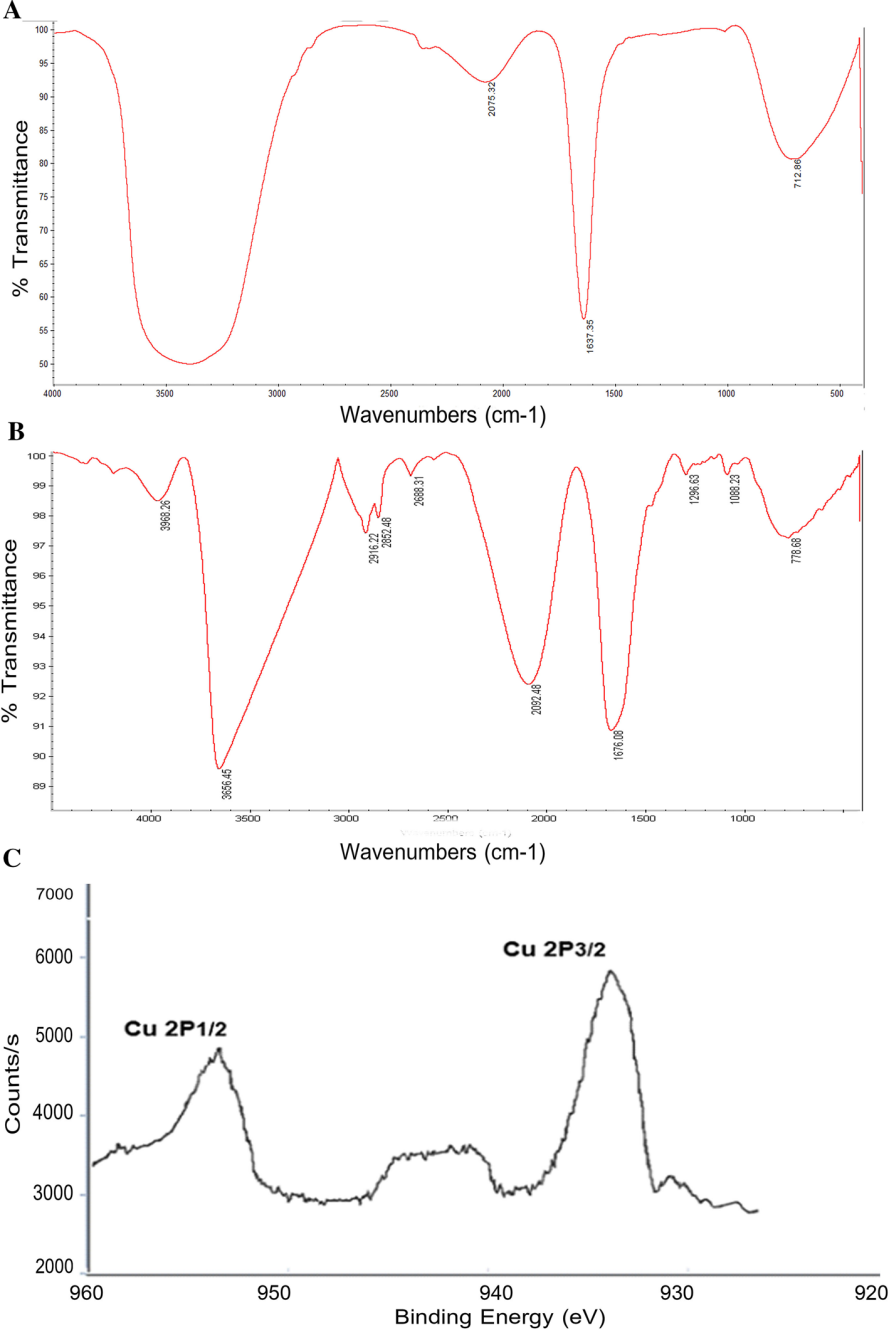
FT-IR spectrum of (**A**) the supernatant of *B. coagulans*, (**B**) BC-CuONPs, (**C**) XPS analysis of BC-CuONPs.

The FTIR can be employed to investigate the surface chemistry of synthesized metallic NPs, to study the role of biological molecules in NP formation, and to analyze various capping agents^61^. As shown in Fig. 4, a strong and broad peak within the frequency range of 3100–3700 cm^−1^ belongs to the tensile adsorption of NH bonds of amide or amine groups in the bacterial protein membrane. Additionally, the adsorption of tensile vibrations of OH groups of alcoholic derivatives and polysaccharides was observed in the FTIR spectrum of the supernatant of *B. coagulans*. The peak 2092 cm^−1^ belongs to the N=C=S group. The peak 1637 cm^−1^ might belong to the C=C bond outside the aromatic ring or the tensile vibrations of the C=O carbonyl bond in the nucleic acid of the cell nucleus. Due to their multiplicity, the peaks associated with the bending vibrations of C–H bonds of alkene and aromatic groups (C=C–H) were observed wildly around the absorption frequency of 712 cm^−1^.

In the FTIR spectrum of reduced CuONPs with bacterial supernatant biomolecules, the peak in the region of 3100–3680 cm^−1^ is attributed to the amide or amine functional group in the bacterial protein membrane or O–H group in Cu(OH)_2_ due to the N–H bond tension. Both 2916 cm^−1^ and 2852 cm^−1^ peaks belong to the aliphatic C–H bond. The peak 2092 cm^−1^ is related to the N=C=S group. The peak 1676 cm^−4^ might belong to the C=C bond out of the aromatic ring or the C=O bond in the nucleic acid of the cell nucleus. The peak 1088 cm^−1^ is associated with the C=O bond in a carboxylic acid. Peak 1296 cm^−1^ is also related to the bending vibration of the C–O–H or C–O ester group in lipids. The FTIR outcomes that verified the existence of the bioactive substances in the supernatant of *B. coagulans*, including amino acids, nucleic acid, carboxylic acid, and lipids, are attributed to the capping and stabilization of BC-CuONPs.

Also, the XPS technique is widely used as a more accurate and reliable technique for the chemical analysis of surface oxides related to than XRD. In Fig. 4C, XPS analysis of BC-CuONPs sample reveals the existence of copper oxide as CuO. The XPS show that the binding energy of Cu 2P3/2 was located at 933.1 eV and the binding energy of Cu 2P1/2 was located at 953.1 eV, showing that copper was found as Cu2+. There is also shake-up satellite peaks located at 941.9 eV.

### Anticancer activity of BC-CuONPs

**Figure 5:**
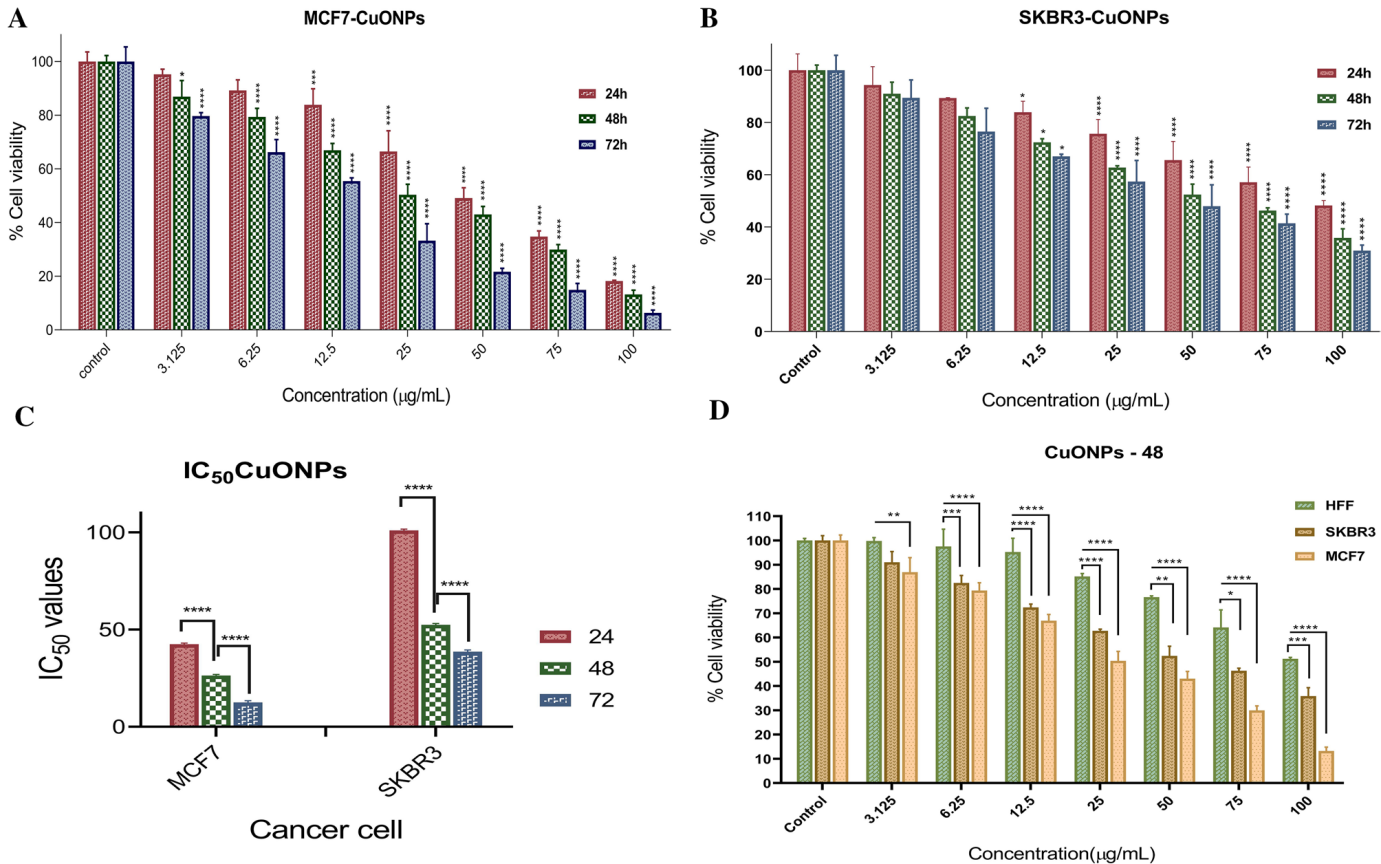
Different concentration cytotoxicity of BC-CuONPs against (**A**) MCF7, (**B**) SKBR3 for 24, 48, and 72 h. (**C**) IC_50_ concentration of BC-CuONPs against MCF7 and SKBR3 cells. (**D**) Comparison of CuONPs-induced cell cytotoxicity in HFF, SKBR3, and MCF7 cell lines after 48 h treatment. (*:*P* < 0.05, **:*P* < 0.01, ***:*P* < 0.001, and ****:*P* < 0.0001).

The MTT assay showed that with increasing BC-CuONPs concentration and treatment time (i.e., 24, 48, and 72 h), the cell viability percentage of MCF-7 and SKBR3 cancer cells significantly decreased, compared to the control group (Fig. 5). The percentage for survived MCF-7 cells reached about 18%, 13%, and 6%, and about 48%, 36%, and 31% for SKBR3 cells after treatment with 100 μg/mL of BC-CuONPs at 24, 48, and 72 h, respectively (*P* < 0.0001) (Fig. 5A,B). Figure 5C shows the IC_50_ values of BC-CuONPs in MCF-7 and SKBR3 cells. The IC_50_ values of BC-CuONPs were 42.86, 26.76, and 13.16 μg/mL for MCF-7 cells and 101.50, 52.90, and 42.86 for SKBR3 cells after 24, 48, and 72 h, respectively. This finding demonstrated that SKBR3 cells required at least a twofold greater concentration of BC-CuONPs to achieve IC_50_ rather than MCF-7 cells. The cytotoxicity results also indicated that the IC_50_ concentration decreased with the elevation of the time period of treatment, and the lowest IC_50_ concentration was relative to 72-h treatments in both cell groups (*P* < 0.001). Biosynthesized CuONPs significantly inhibited the proliferation of MCF-7 and SKBR3 breast cancer cells in a time- and dose-dependent manner. According to cytotoxicity results, biogenic BC-CuONPs showed greater anti-proliferative activity against MCF-7 cells than SKBR3 cells.

The MTT results of HFF cells proved very low toxicity of BC-CuONPs against normal cells. The results showed that the highest concentration of BC-CuONPs (100 μg/mL) induced 51% cytotoxicity after the 48-h treatment in HFF normal cells (Fig. 5D). Therefore, the breast cancer cells were considerably more susceptible to the biogenic CuONPs than HFF normal cells.

**Figure 6:**
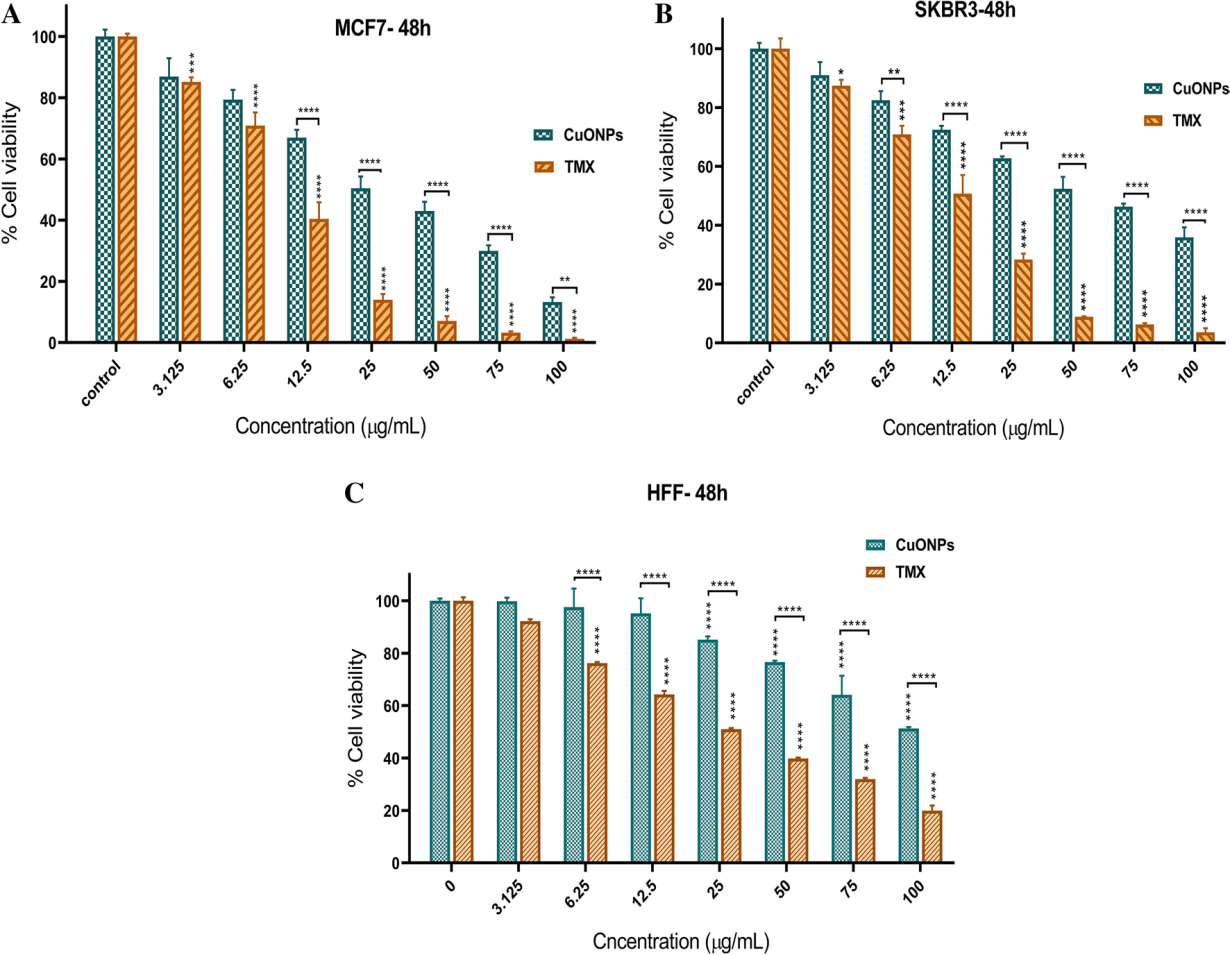
Comparison of tamoxifen and CuONPs-induced cell cytotoxicity in (**A**) MCF7, (**B**) SKBR3, and (**C**) HFF cells after 48 h treatment. (*:*P* < 0.05, **:*P* < 0.01, ***:*P* < 0.001, and ****:*P* < 0.0001).

The cytotoxic activity of tamoxifen was compared to BC-CuONPs against MCF-7, SKBR3, and HFF cells (Fig. 6). These results revealed that tamoxifen exerted more cytotoxicity against MCF-7, SKBR3, and HFF normal cells than BC-CuONPs (Fig. 6A–C). A comparison of the cytotoxicity results of tamoxifen and BC-CuONPs demonstrated that biogenic BC-CuONPs had a much less toxic effect on HFF normal cells than tamoxifen (Fig. 6C). One of the most important disadvantages of tamoxifen is its high toxicity effects on normal cells, as demonstrated in this study. The results of the current study showed very low cytotoxicity of biosynthesized BC-CuONPs against HFF normal cells, demonstrating their biocompatibility.

There are various studies that indicate the anticancer activity of CuONPs against different cancer cell lines. Furthermore, the synthesized CuONPs were screened for anticancer activity in the human colon cancer cell line (HCT-116) by MTT assay. Gnanavel et al.’s study and Obaid et al.’s investigation indicated that the synthesized CuONPs show great anticancer effects on HCT-116 and HT-29 cancer cell lines with the IC_50_ value of 40 μg/mL for 24 h, which was close to the current study’s findings^62,63^. Moreover, an investigation showed that the cell viability of HepG2 cell lines had a significant decrease in time- and dose-depended manner after treatment with biosynthesized CuONPs using *Momordica cochinchinensis*^64^. Another study indicated that the IC_50_ values of biosynthesized CuONPs using *Annona muricata* extract for AMJ-13 and MCF-7 cells were 17.04 and 18.92 µg mL^−1^, respectively, which demonstrates that the uptake of CuONPs by cancer cells could trigger apoptosis^6^.

Sathiyavimal et al. worked on the anticancer activity of biosynthesized CuONPs using *Abutilon indicum* leaves and showed significant cytotoxicity of these NPs against MDA-MB-231 breast cancer cell line^65^. Chinnathambi et al. studied novel copper nanoparticles (CuNPs) using *Allium noeanum* leaf to treat endometrial cancer. The IC_50_ values of *A. noeanum* leaf aqueous extract and CuNPs against the HEC-1-B cell line were 548 and 331 µg/mL, respectively; the IC_50_ values against HEC-1-A cell line were 583 and 356 µg/mL, respectively; the IC_50_ values against KLE cell line were 609 and 411 µg/mL, respectively; the IC_50_ values against Ishikawa cell line were 560 and 357 µg/mL, respectively^66^. The difference between the IC_50_ of the aforementioned study with the present study could be due to the synthesis method and different cancer cell types.

Another study demonstrated that synthesized CuONPs using *Lactobacillus plantarum* exert a cytotoxic effect on the human liver cell line WRL-68 and the MCF-7 cell line with the elevation of CuONPs concentration. The IC_50_ values for WRL-68 and MCF-7 cells were 153.0 and 63.64 μg/mL, respectively^67^. Karlsson et al. revealed that CuONPs have the potential to exert serious damage to DNA and have significant cytotoxicity against A549 cancer cells^68^. Previous studies emphasize that CuONPs exert an anticancer effect against turmeric cells via the induction of apoptosis and generation of ROS^69,70^.

Salami et al. showed that tamoxifen could induce apoptosis in MCF-7 and MDA-MB-231 cells^71^. An investigation also indicated that tamoxifen could induce the sustained activation of ERK1/2 in ER-positive breast cancer cell lines (i.e., MCF-7 and T47D), which resulted in cell death^72^. These observations are in agreement with the findings of the current study, and the small observed variation could be due to different reducing agents in NP synthesis and different cell lines.

### Induction of apoptosis and arrest of cell cycle by BC-CuONPs in breast cancer cells

**Figure 7:**
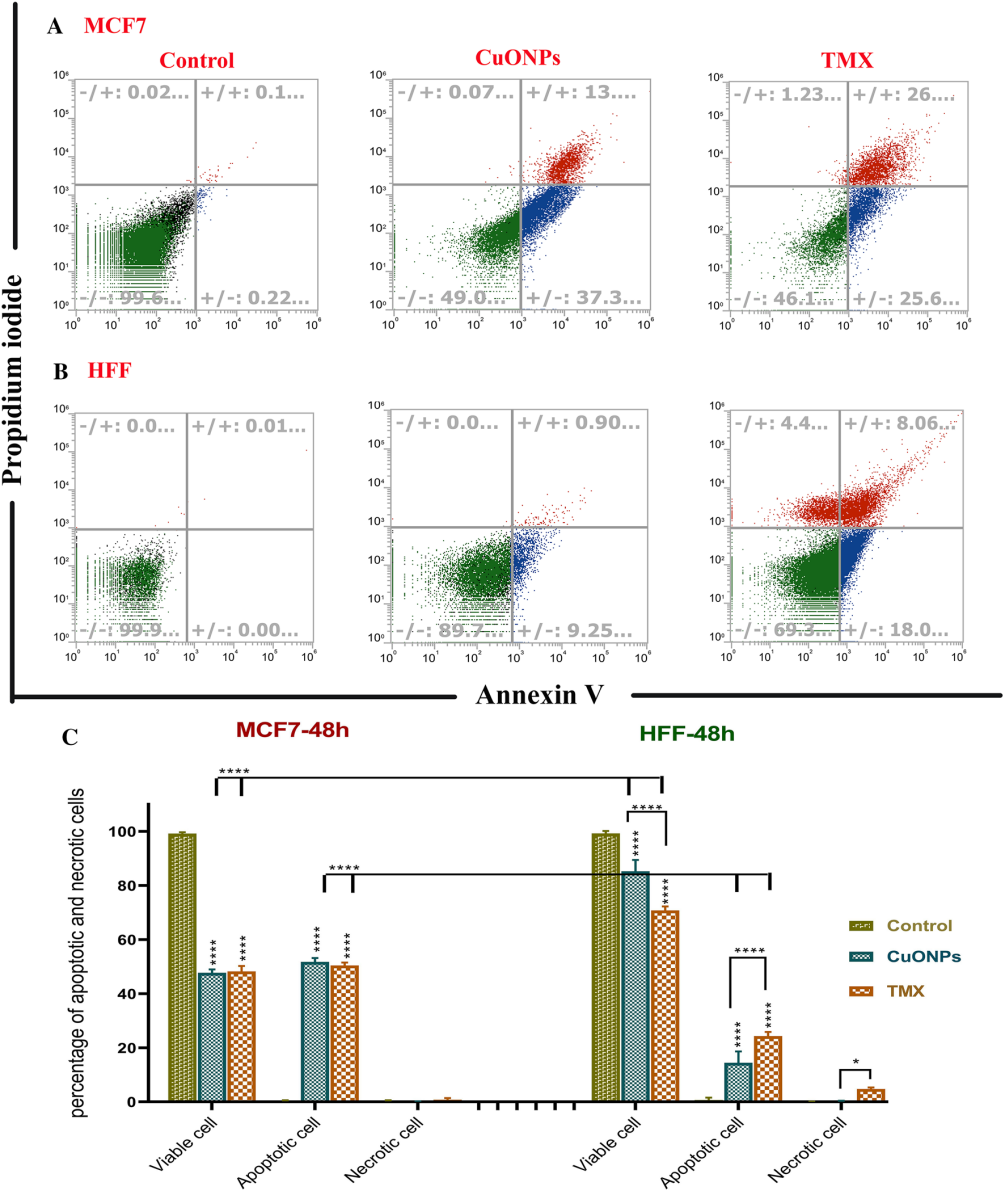
Apoptosis rate in (**A**) MCF7 and (**B**) HFF cells treated with IC_50_ concentrations of BC-CuONPs and tamoxifen (TMX). (**C**) Comparison of apoptosis and necrosis rate in MCF7 and HFF cells treated with IC_50_ concentrations of BC-CuONPs and tamoxifen after 48 h treatment. *:*P* < 0.05, ****:*P* < 0.0001.

**Figure 8:**
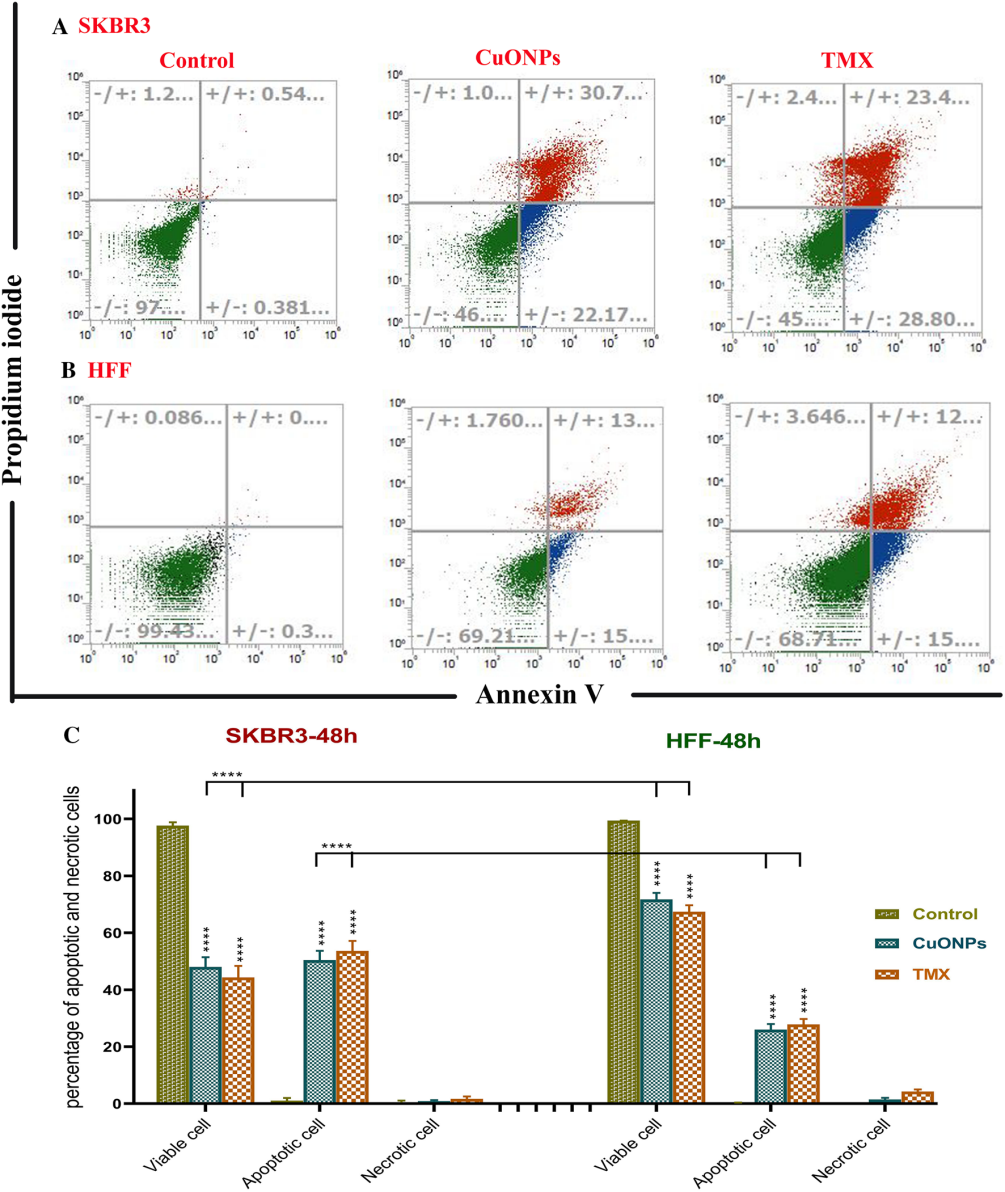
Apoptosis rate in (**A**) SKBR3 and (**B**) HFF cells treated with IC_50_ concentrations of BC-CuONPs and tamoxifen (TMX). (**C**) Comparison of apoptosis and necrosis rate in SKBR3 and HFF cells treated with IC_50_ concentrations of BC-CuONPs and tamoxifen after 48 h treatment. ****:*P* < 0.0001.

The percentage of early apoptosis, late apoptosis, and necrosis were estimated at 37.3%, 13%, and 0.07% for CuONPs treatment and 25.6%, 26%, and 1.23% for tamoxifen in MCF-7 cells, respectively (Fig. 7A–C). The aforementioned percentages were also estimated at 22.17%, 30.17%, and 1% for CuONPs and 28.80%, 23.4%, and 2.4% for tamoxifen in SKBR3 cell line, respectively (Fig. 8A–C). The apoptosis rate in BC-CuONPs-treated HFF cells was lower than in tamoxifen-treated cells. The percentage of early and late apoptotic cells was also significantly reduced in HFF cells, compared to MCF-7 and SKBR3 cancer cells (*P* < 0.001) (Figs. 7C, 8C). On the other hand, the apoptotic rate was remarkably higher in MCF-7 and SKBR3 cells treated with both BC-CuONPs and tamoxifen. Nevertheless, the data demonstrated that the apoptotic effect of BC-CuONPs against both cancer cell lines was comparable to tamoxifen results (Figs. 7C, 8C). This study also showed that tamoxifen could induce remarkable necrosis (4.4%) in normal cells (*P* < 0.05); nevertheless, BC-CuONPs showed no necrosis in HFF cells, which is a suitable property of NPs relative to the drug (Figs. 7, 8).

An investigation indicated that the exposure of B16F10 cells to biosynthesized CuNPs at IC_50_ led to about 50% induction in apoptosis^73^. An in vivo study also revealed that significant tumor suppression and an increased ratio of apoptotic over live tumor-initiating cells were observed in mice treated with CuONPs, compared to control animals^15^. Chakraborty et al. demonstrated that CuONPs could stimulate apoptosis in the A-375 cell line (skin cancer cells) via reducing cell membrane rigidity, cleavage of DNA, condensation of chromosomal, arrest of the cell cycle in the G2/M phase, the mitochondrial membrane depolarization, and activation of the caspase-mediated intrinsic pathway. Further investigation revealed that the cancer cell viability and the mitochondrial membrane potential were decreased in the exposure of CuONPs, which resulted in enhancement in the apoptosis rate and induction of oxidative stress^37,74^.

In another investigation, the biosynthesized CuONPs using *Ziziphus zizyphus* leaf extract were tested for their anticancer activity on human renal carcinoma A498 cells, which indicated the significant cytotoxicity of CuONPs and elevated ROS production followed by induced apoptosis in A498 cells. Induced apoptosis was verified by the upregulation of *BID, BAX, CASP3,* and *CASP9* and the downregulation of *BCL2* gene expression in A498 cells treated with biosynthesized COuNPs^75^.

**Figure 9:**
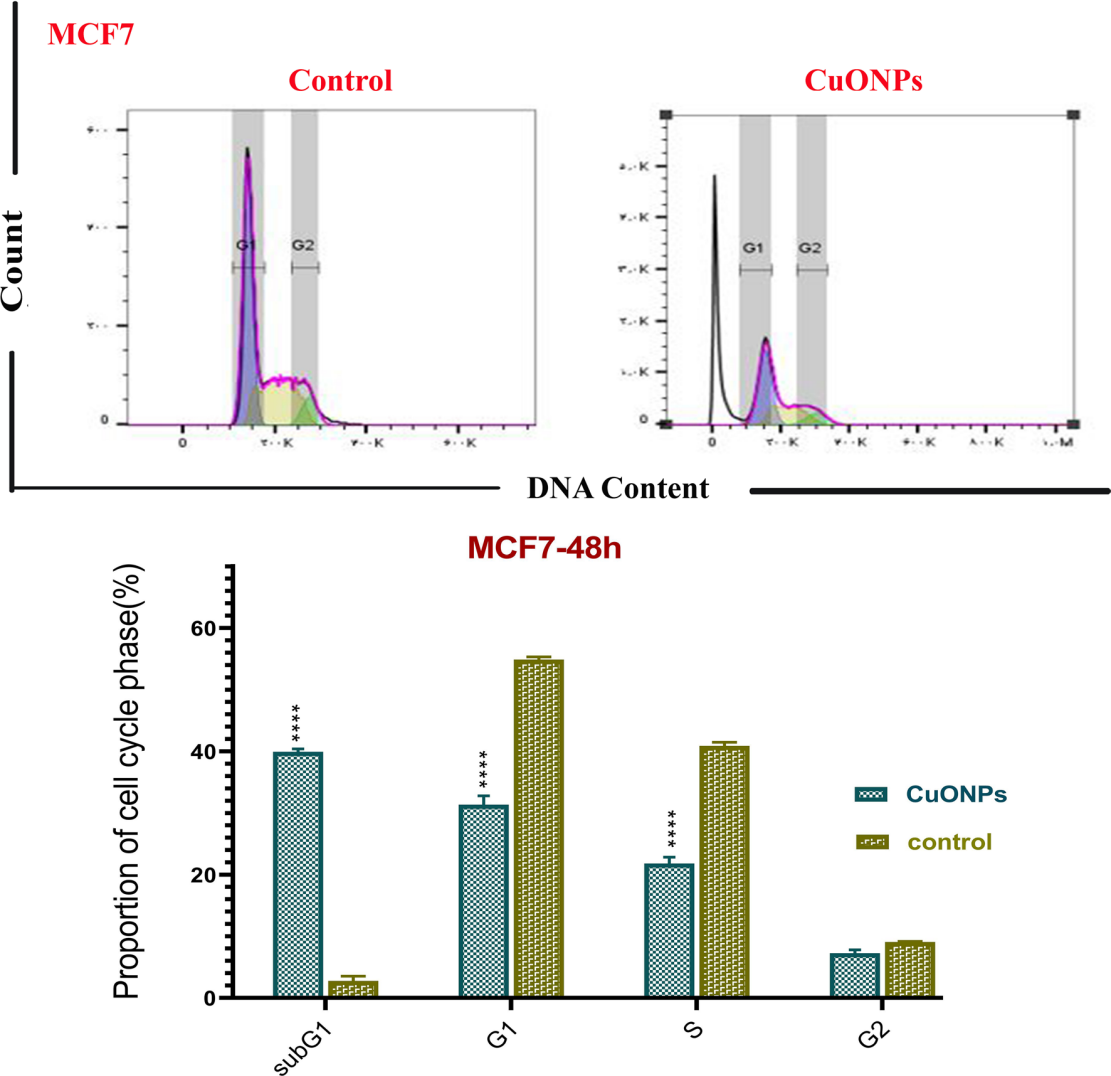
Cell cycle arrest in MCF7 cells treated with IC_50_ concentration of BC-CuONPs. Comparison of cell cycle arrest of different phases in MCF7 cells treated with IC_50_ concentrations of BC-CuONPs after 48 h treatment. ****:*P* < 0.0001.

**Figure 10:**
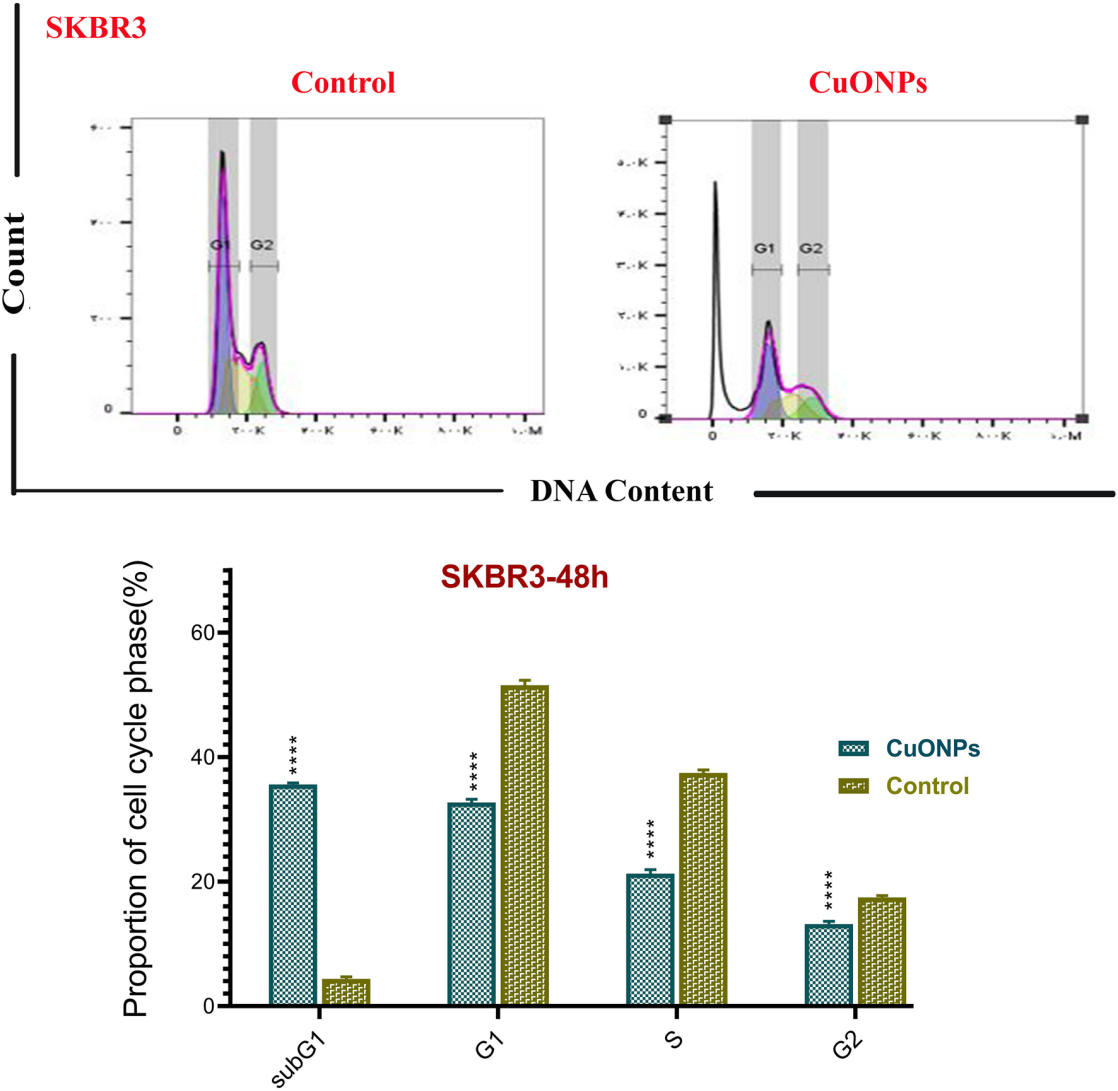
Cell cycle arrest in SKBR3 cells treated with IC_50_ concentrations of BC-CuONPs. Comparison of cell cycle arrest of different phases in SKBR3 cells treated with IC_50_ concentrations of BC-CuONPs after 48 h treatment. ****:*P* < 0.0001.

The cell cycle is a four-phase mechanism in which a cell becomes bigger (G1), replicates its DNA (S), and is ready for mitosis (G2) and mitosis (M). Anti-mitotic substances prevent cells from progressing through the phases of mitosis. As a result, numerous cells do not initiate the G1 phase of the cell cycle because they restrict at the initiation stage. Such cells remain in a detached phase recognized as the sub-G1 phase. The current study investigated the impacts of BC-CuONPs on MCF-7 and SKBR3 cells using flow cytometry during their cycle (Figs. 9, 10). The steady impact of BC-CuONPs was demonstrated by cells entering the sub-G1 phase in each examined cell line (MCF-7 cells: 40%; SKBR3 cells: 35.60%).

Cell cycle regulation is one of the most important modifications in the growth of cancer cells. The essential characteristics of checkpoints, cyclins, and cyclin-dependent kinases alter at various phases of the cell cycle and govern cell cycle development. Due to cellular damage and stress signaling, the cell cycle might be stopped at specific phases, rendering the checkpoints faulty^76^. Benguigui et al. demonstrated that CuONPs could stimulate the apoptosis of tumor-initiating cells of pancreatic tumors and induce their arrest in the sub-G1 phase^15^. Another investigation discovered that CuONPs synthesized using *Olea europaea* could be efficient in inducing dose-dependent cell cycle arrest and apoptosis via the stimulation of the apoptotic pathway in breast cancer (AMJ-13) and ovarian cancer (SKOV-3) cells^77^.

Ali et al.’s study indicated that after the exposure of MCF-7 cells to 100 μg/mL of biosynthesized CuONPs using *Eucalyptus globulus* leaf extract, the G1 population decreased significantly. Simultaneously, the guaranteed decline in the G2/M population was observed with an enhancement in the sub-G1 phase, demonstrating that cells could not repair DNA damage and finally inserted the apoptotic or necrotic phase^78^. The current survey indicated the high potential of CuONPs to induce cell arrest in the sub-G1 phase.

### Effect of BC-CuONPs on expression of pro-apoptotic, ani-apoptotic, and cell cycle related genes in breast cancer cells

**Figure 11:**
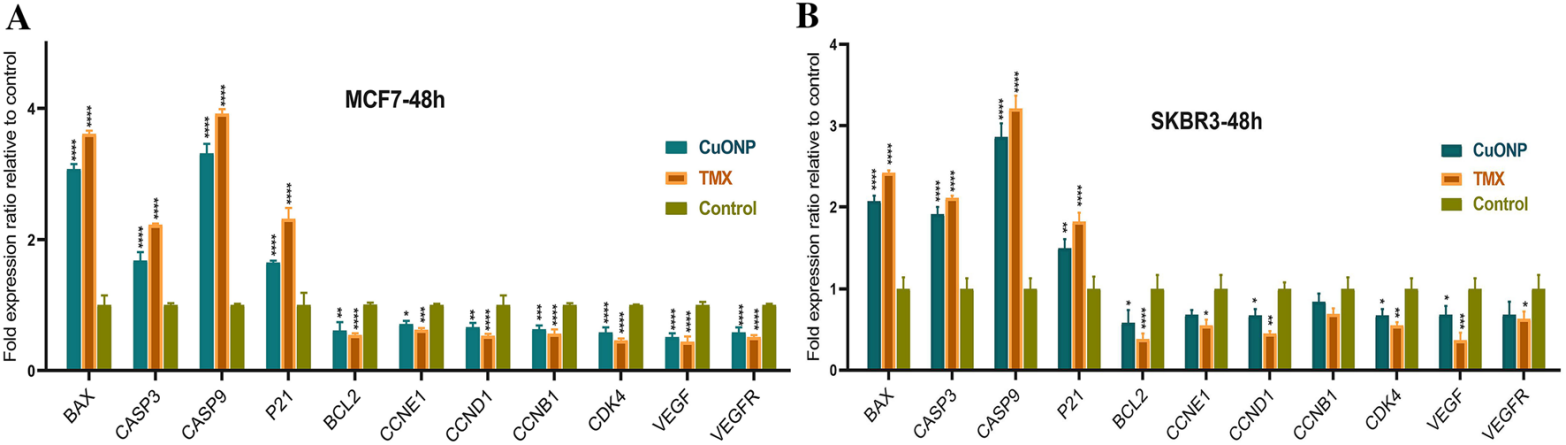
*BAX, BCL2, CASP3, CASP9, P21, VEGF, VEGFR, CCND1, CCNE1, CCNB1, CDK4*, and *ACTB* expression levels in (**A**) MCF7 and (**B**) SKBR3 cell lines treated with IC_50_ concentration of BC-CuONPs and tamoxifen (TMX) after 48 h. (*:*P* < 0.05, **:*P* < 0.01, ***:*P* < 0.001, and ****:*P* < 0.0001).

Figure 11 depicts the effects of BC-CuONPs and tamoxifen on the mRNA expression levels of *CASP3, CASP9, BAX, BCL2, P21, VEGF, VEGFR, CCND1, CCNE1, CCNB1,* and *CDK4* in two SKBR3 and MCF-7 cells. These genes might be divided into three chief categories, pro-apoptotic, anti-apoptotic, and angiogenic.

According to Fig. 11, the expression levels of some pro-apoptotic genes (including *BAX, CASP3,* and *CASP9*) and *P21* in MCF-7 cells had statistically significant up-regulation, following treatment with CuONPs and tamoxifen, compared to the control group (*P* < 0.0001). A significant down-regulation was also observed in the expression of *BCL2* (*P* < 0.01, *P* < 0.0001), *CCNB1* (*P* < 0.001, *P* < 0.0001), *CCND1* (*P* < 0.01, *P* < 0.0001), *CCNE1* (*P* < 0.05, *P* < 0.001), *CDK4* (*P* < 0.0001), *VEGF* (*P* < 0.0001), and *VEGFR* (*P* < 0.0001) genes in MCF-7-treated cells with both BC-CuONPs and tamoxifen.

In SKBR3 cells, the expression of *BAX, CASP3*, *CASP9* (*P* < 0.0001), and *P21* (*P* < 0.01) genes also had remarkable elevation, and the expression levels of *BCL2, CDK4, CCND1,* and *VEGF* (*P* < 0.05) genes showed significant down-regulation, following treatment with CuONPs. The tamoxifen could not only decline the expression of *BCL2* (*P* < 0.0001), *CDK4* (*P* < 0.01), *CCND1* (*P* < 0.01), and *VEGF* (*P* < 0.001) genes but also decrease the expression of *VEGFR* and *CCNE1* (*P* < 0.05). However, only tamoxifen could considerably decline the expression of *VEGFR* (*P* < 0.0001) and *CCNE1* genes (*P* < 0.05). The data indicated that the alteration of induced gene expression in cells treated with BC-CuONPs was comparable to the tamoxifen-treated group.

The proteins of the BCL2 and caspases family represent an important regulator for apoptotic cell death. BCL2 and BAX are important regulators of mitochondrial membrane penetrability, mitochondrial performance, and cytochrome c secretion^79^. Cytochrome c might also interact with apoptotic protease activating factor-1, resulting in the formation of an apoptosome and the activation of caspase 9. Caspase 9 might effectively cleave and activate caspase 3 and caspase 7^80^. Caspase 3 is coordinated in the breakdown of cellular components, such as DNA breakage or cytoskeletal protein breakdown^81^.

The P21 functions as a negative regulator of P53-dependent apoptosis and can be activated by both P53-dependent and P53-independent pathways. The P21 plays a key role in the changes in gene expression involved in cell cycle development, DNA repair, and apoptosis control, including the E2F family, nuclear factor kappa-light-chain-enhancer of activated B cells (NF-κB), C-myc, STAT, and p300/CPB^82^. An in vitro study indicated that CuONPs treatment increased the expression of the *P53* tumor suppressor gene, and an elevation in the *BAX*/*BCL2* ratio revealed that a mitochondria-mediated mechanism is engaged in CuONPs-induced programmed cell death^17^. The findings of another investigation showed that the expression levels of *P21*, *BAX*, and *CASP3* were considerably elevated, and *BCL2* dramatically declined in CuONPs-treated SKBR3 cells as compared to normal cells^83^.

Another investigation indicated that CuONPs could dramatically increase the mRNA expression of *P53* and *P21* in A549 cells in comparison to control^84^. Therefore, the over-expression of *BAX, BCL2, CASP3, CASP9, P21,* and *P53* after the treatment with CuONPs presented their potential to operate as a great anticancer agent. The CuONPs in Phull et al.’s study were synthesized using *Undaria pinnatifida*-derived fucoidan as a capping and reducing agent and indicated that the expression of BCL2 protein decreased. Moreover, the expression of BAX, cytochrome c, cleaved CASP-3 (cleaved caspase-3), and cleaved poly (ADP-ribose) polymerase proteins elevated in CuONPs-treated HeLa cell lines^85^.

A study revealed that the treatment of green synthesized CuONPs using *Azadirachta indica* could enhance toxicity, elevate ROS production, and increase DNA fragmentation in cancer MCF7 and HeLa cells. Furthermore, an elevation in the level of apoptotic protein markers, such as BAX, Caspase 9, Caspase 8, Caspase 3, P38, and cytochrome c, was observed in cancer cells after the treatment with CuONPs^58^.

The *VEGF* and *VEGFR* genes have been demonstrated to serve important functions in both physiological angiogenesis and pathological angiogenesis (i.e., cancer progression). Cyclin is a protein category that regulates cell cycle development by activating cyclin-dependent kinase (CDK) enzymes or groups of enzymes essential for cell cycle formation^86^. The cyclin D1 proto-oncogene regulates G1 to S phase transition in various cell lines^87^. Cyclin E1 up-regulation not only stimulates tumorigenesis by boosting cell cycle transition but also promotes the production of associated proteins, resulting in chemoresistance^88^. Furthermore, the cyclin B1/CDK1 complex regulates the G2/M phase transition and is required for mitosis to begin. A malfunctioning G2/M checkpoint will result in genomic disruption and cancer initiation. As a result, the abnormal cyclin B1 and CDK1 expression encourage excessive cell proliferation and tumor progression^89^.

The present study’s findings are in line with those of other studies; nevertheless, the elevated concentration of CuONPs caused a significant reduction in *VEGF* expression and elevated apoptosis in 4T1 cancer cells^90^. In a previous investigation conducted by Luo et al.^91^, a dose- and time-dependent reduction was observed in the levels of cyclin A and cyclin B1 in HaCaT cells treated with CuONPs. There was no sufficient experiment associated with the effects of CuONPs on other cyclin genes’ expression. However, the present study reported that CuONPs could elevate pro-apoptotic genes’ expression and decline anti-apoptotic, angiogenic genes, and cell cycle development-associated genes’ expression in cancerous cell lines.

### Induction of ROS generation by BC-CuONPs in breast cancer cells

**Figure 12:**
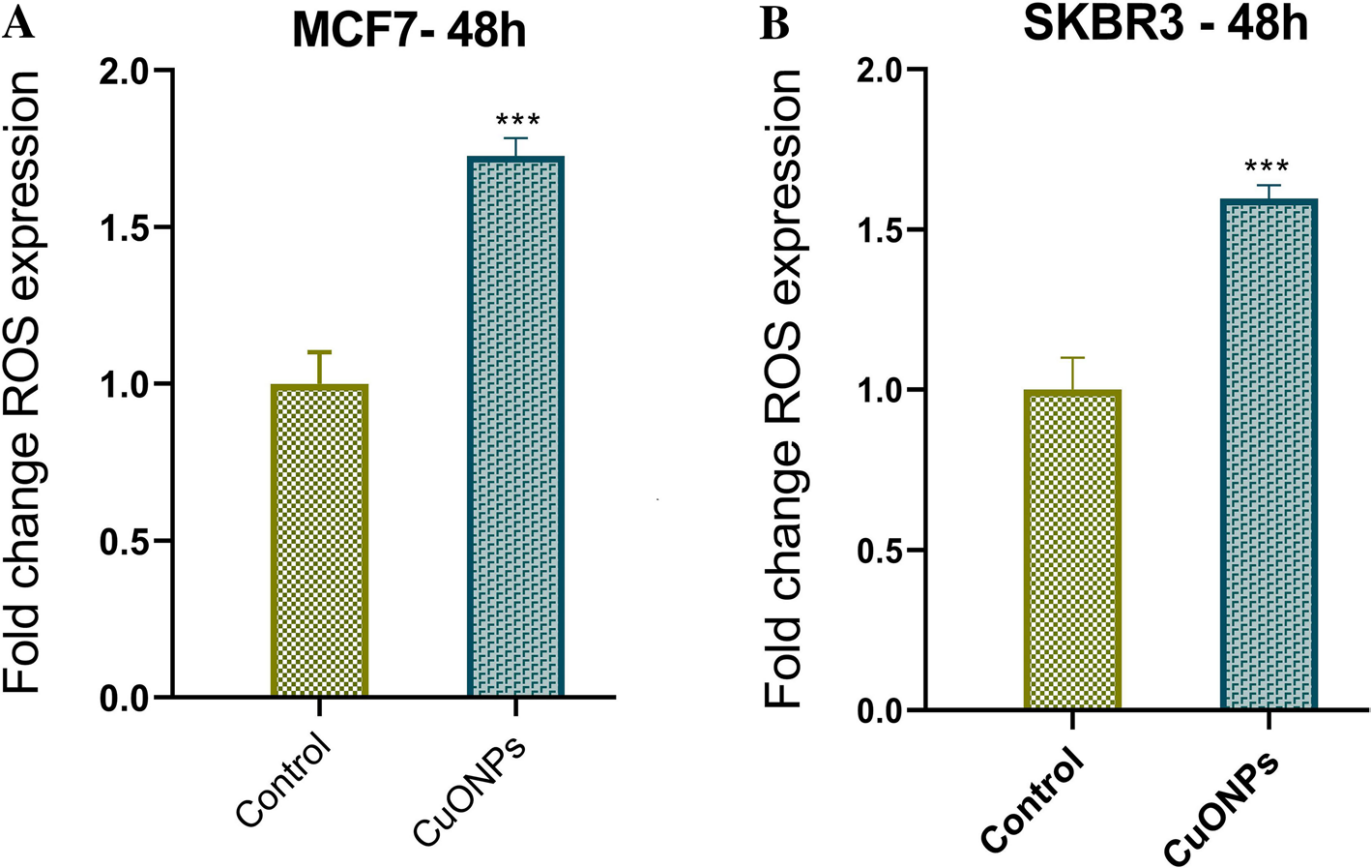
ROS levels in (**A**) MCF7 and (**B**) SKBR3 cell lines treated with IC_50_ concentration of BC-CuONPs after 48 h. ***:P < 0.001.

The ROS has been shown in various investigations to have an important role in the inhibitory behavior of tumor-induced immunosuppressive cells. The intracellular ROS content of control and CuONPs-treated MCF-7 and SKBR3 cells is depicted in Fig. 12. As demonstrated in Fig. 12, approximately 1.8-fold and 1.6-fold enhancement was observed in the ROS content of MCF-7 and SKBR3 cells after the 48-h treatment, compared to the control, respectively. The CuONPs have been shown to have cytotoxic and genotoxic effects on living organisms by producing ROS^17,92^. Various experiments indicated that CuONPs could induce ROS generation in different cancer cells, including HepG2^93^, SK-Hep-1^12^, K562^17^, tumor-initiating cells-enriched PANC1^15^, and MCF-7 cells^78^. In another study, the incubation of HepG2 cancer cells with biosynthesized CuONPs at concentrations of 25–100 μg/mL for 48 h resulted in the elevation of ROS generation, compared to control cells^64^.

Excessive ROS might potentially be employed to effectively demolish cancer cells due to their apoptosis-inducing capabilities. Furthermore, it is claimed that cancer cells might be more susceptible to ROS generation than healthy cells, such that enhanced oxidative stress caused by external ROS formation might be exploited as an anticancer therapeutic technique to preferentially destroy cancer cells while not damaging normal cells^94,95^. Malignant transformation is caused by raised ROS levels and down-regulation of cellular antioxidant enzyme systems via many molecular targets, including NF-κB and nuclear factor erythroid 2-related factor 2^96^. These important components control signaling pathways that create an inflammatory milieu and prevent tumor growth, angiogenesis, and metastasis, all of which together promote the beginning, development, and spread of malignant neoplasms^97^.

### Effect of BC-CuONPs on wound healing

**Figure 13:**
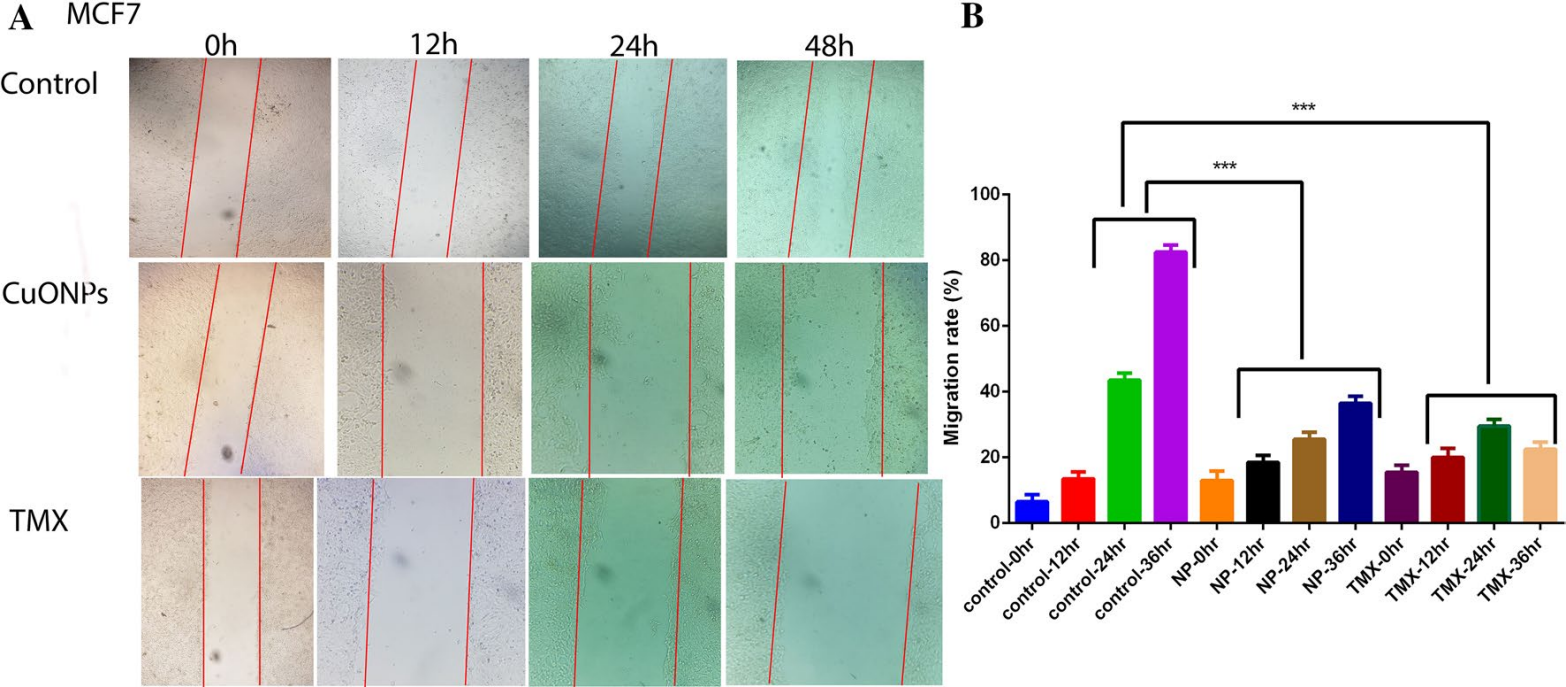
(**A**) Scratch assay in MCF7 cell lines treated with IC_50_ concentrations of BC-CuONPs and tamoxifen (TMX) after 48 h. (**B**) Comparison of scratch tests in controls and MCF7 cells treated with IC_50_ concentrations of BC-CuONPs and tamoxifen in 12, 24, and 36 h treatments. ***:*P* < 0.001.

A scratch wound experiment was performed on MCF-7 cells to evaluate each drug’s anti-metastatic characteristics. To test the growth inhibitory impact of BC-CuONPs and tamoxifen, a wound-healing experiment was performed (Fig. 13). Both therapies had a strong inhibitory impact on cell migration. The cells’ migration area (toward the originally scratched center line from the borderline) was monitored. In the control group, cell migration boosted significantly with increasing time. However, the treatment with BC-CuONPs and tamoxifen caused a significant reduction in cell migration, compared to the control group, during 36 h (*P* < 0.001). The biogenic BC-CuONPs demonstrated high anti-metastatic activity as much as tamoxifen. According to the findings of a study, A549 cells treated with AgNPs and cisplatin invaded and migrated; however, the anti-migratory impact of AgNPs on A549 cells was much more significant than on cisplatin-treated cells and the control^53^. The aforementioned findings showed that the tailored CuONPs of this study might be employed successfully in the treatment of breast cancer metastases.

## Conclusion

In the last several years, biological NPs production procedures by various microorganisms that are harmless, economical, and ecologically friendly have expanded. *Bacillus coagulans* species has been reported as a lactic acid-producing, Gram-positive, non-pathogenic, and endospore-producing probiotic that is resistant to heat and pH. Considering that in the supernatant of bacteria, there are diverse biomolecules, such as enzymes, vitamins, polysaccharides, and amino acids, that reduce metal ions to NPs, and this method is a green and ecologically friendly way, this study reported the effective fabrication of CuONPs utilizing *B. coagulans* supernatant (BC-CuONPs). The successful synthesis of CuONPs was carried out using *Bacillus coagulans*, and excellent in vitro anticancer and apoptotic activity was shown against human breast cancer cells.

**Figure 14:**
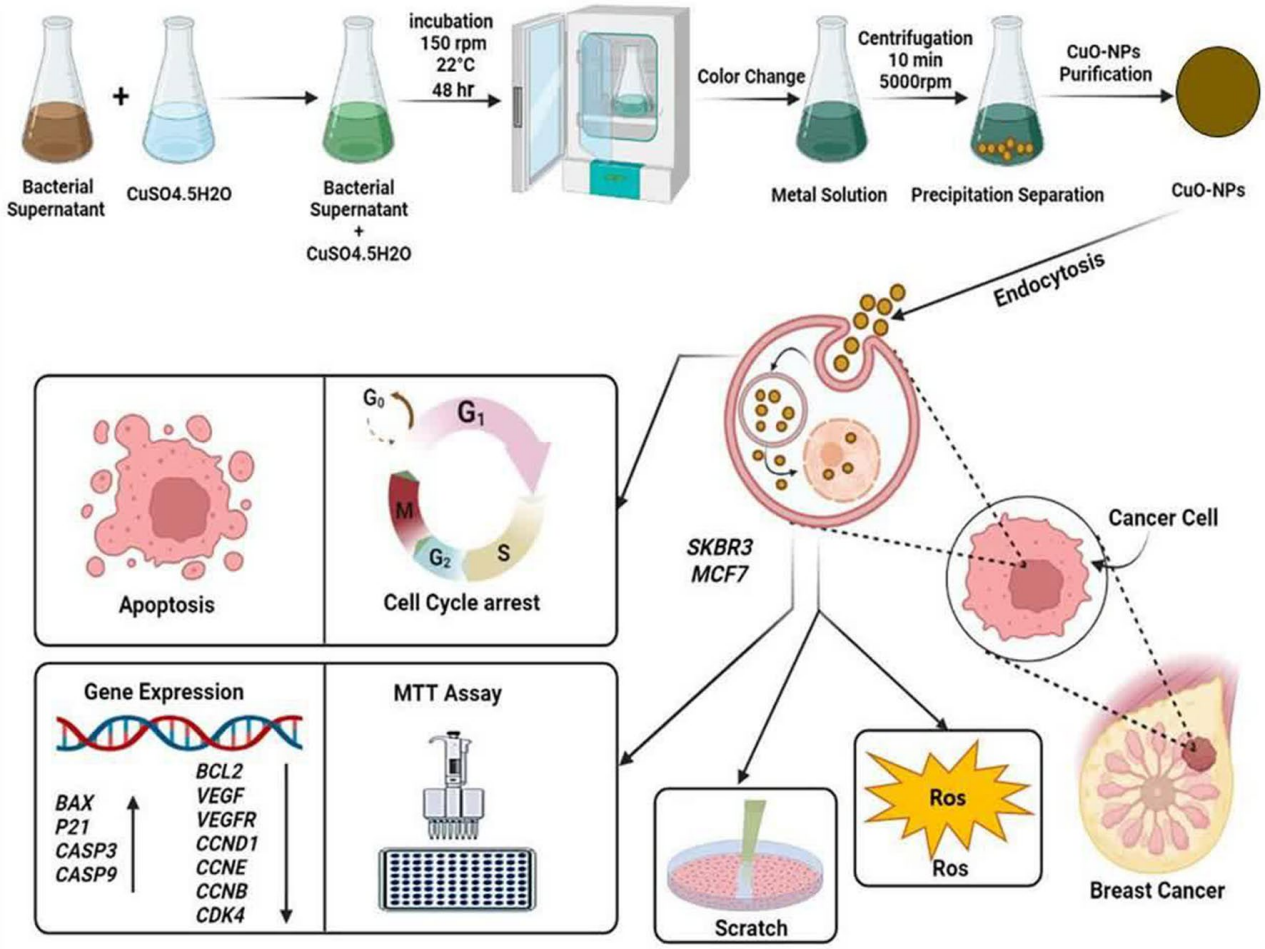
Biogenic synthesis of CuONPs using *Bacillus coagulans*, cellular uptake, ROS generation, apoptosis induction, and cell cycle arrest in breast cancer cells.

The biogenic BC-CuONPs demonstrated strong cytotoxic activity and apoptosis induction against breast cancer cells, comparable to tamoxifen. Furthermore, BC-CuONPs might suppress breast cancer cell metastasis and increase ROS generation and cell cycle arrest in the sub-G1 phase (Fig. 14). Moreover, a low cytotoxic effect was observed in HFF normal cells, which demonstrated the biocompatibility of BC-CuONPs. Further in vivo experiments could elucidate the potential of BC-CuONPs to be applied as a medicinal agent in cancer therapy.

## Data Availability

The datasets used and/or analyzed during the current study are available from the corresponding author on reasonable request.
